# Proteoglycans and Immunobiology of Cancer—Therapeutic Implications

**DOI:** 10.3389/fimmu.2019.00875

**Published:** 2019-04-24

**Authors:** George Tzanakakis, Monica Neagu, Aristidis Tsatsakis, Dragana Nikitovic

**Affiliations:** ^1^Laboratory of Anatomy-Histology-Embryology, School of Medicine, University of Crete, Heraklion, Greece; ^2^Immunology Department, “Victor Babes” National Institute of Pathology, Bucharest, Romania; ^3^Pathology Department, Colentina Clinical Hospital, Bucharest, Romania; ^4^Laboratory of Toxicology, University of Crete, Heraklion, Greece

**Keywords:** proteoglycans, immunobiology, cancer, extracellular matrix, remodeling

## Abstract

Disparity during the resolution of inflammation is closely related with the initiation and progression of the tumorigenesis. The transformed cells, through continuously evolving interactions, participate in various exchanges with the surrounding microenvironment consisting of extracellular matrix (ECM) components, cytokines embedded in the ECM, as well as the stromal cells. Proteoglycans (PGs), complex molecules consisting of a protein core into which one or more glycosaminoglycan (GAG) chains are covalently tethered, are important regulators of the cell/matrix interface and, consecutively, biological functions. The discrete expression of PGs and their interacting partners has been distinguished as specific for disease development in diverse cancer types. In this mini-review, we will critically discuss the roles of PGs in the complex processes of cancer-associated modulation of the immune response and analyze their mechanisms of action. A deeper understanding of mechanisms which are capable of regulating the immune response could be harnessed to treat malignant disease.

## Introduction

Cancer initiation is a multi-faceted process with a contribution of genetic, metabolic and environmental factors. Tumorigenesis is closely associated with chronic inflammation, with approximately 20% of cancer incidences being directly correlated to chronic infections (1). Indeed, all tumors independently of etiology are distinguished by an early inflammatory milieu and characterized by discrete interactions with the immune system at all stages of disease progression (2, 3). During malignant transformation, cells obtain complex biological characteristics correlated with more efficient survival, invasion, metastasis and the ability to evade the immune response. The transformed cells, through continuously evolving interactions, communicate with and alter the surrounding microenvironment consisting of extracellular matrix (ECM) components, cytokines embedded in the ECM, and the stromal cells (e.g., fibroblasts, endothelial cells, adipocytes, and immune cells) (4, 5). The resulting ECM remodeling crucially contributes to the abnormal tumor inflammatory pattern (5, 6).

Proteoglycans (PGs) are complex molecules consisting of a protein core into which one or more glycosaminoglycan (GAG) chains are covalently tethered. The bound GAGs can be the heparan sulfate (HS), the chondroitin sulfate/dermatan sulfate (CS/DS), or the keratan sulfate (KS) type. In mammalian cells PGs are associated to the plasma membranes, released into the ECM or intracellularly localized. Presently, 45 PGs have been identified with each member characterized by immense alterability attributed to the modifications of the protein core and by the type and different stoichiometry of the GAG chain substitutions (7, 8). Thus, PGs have highly specific and multifaceted biological roles, including: (i) contributing to ECM superstructure (9); (ii) defining ECM biochemical and physicochemical properties (9); (iii) acting as receptors of diverse responsiveness as well as a pool of various biologic effectors such as growth factors (10–12).

It is well-established that malignant tumors have discrete PG expression profiles, which are immediately correlated with their behavior and differentiation status. Thus, epithelial tumors exhibit a different PG characterization as compared to mesenchymal tumors (13, 14). Proteolytic cleavage of PGs, due to the action of matrix metalloproteinases (MMPs), cathepsins, and bone-morphogenetic protein-1, can release bioactive fragments or matrikines with roles in the propagation of tumorigenesis separate from that of parent molecules (15). Importantly, PGs are highly implicated in the processes of cancer-associated inflammation (16, 17). Indeed, PGs are suggested to modulate key events respective to both innate and adaptive immunity (18, 19).

In this review, we provide a critical overview of PGs' roles in the inflammatory cancer milieu and consecutively extrapolate to potential options for the development of targeted cancer therapies.

## Immunobiology of Cancer

**Figure 1:**
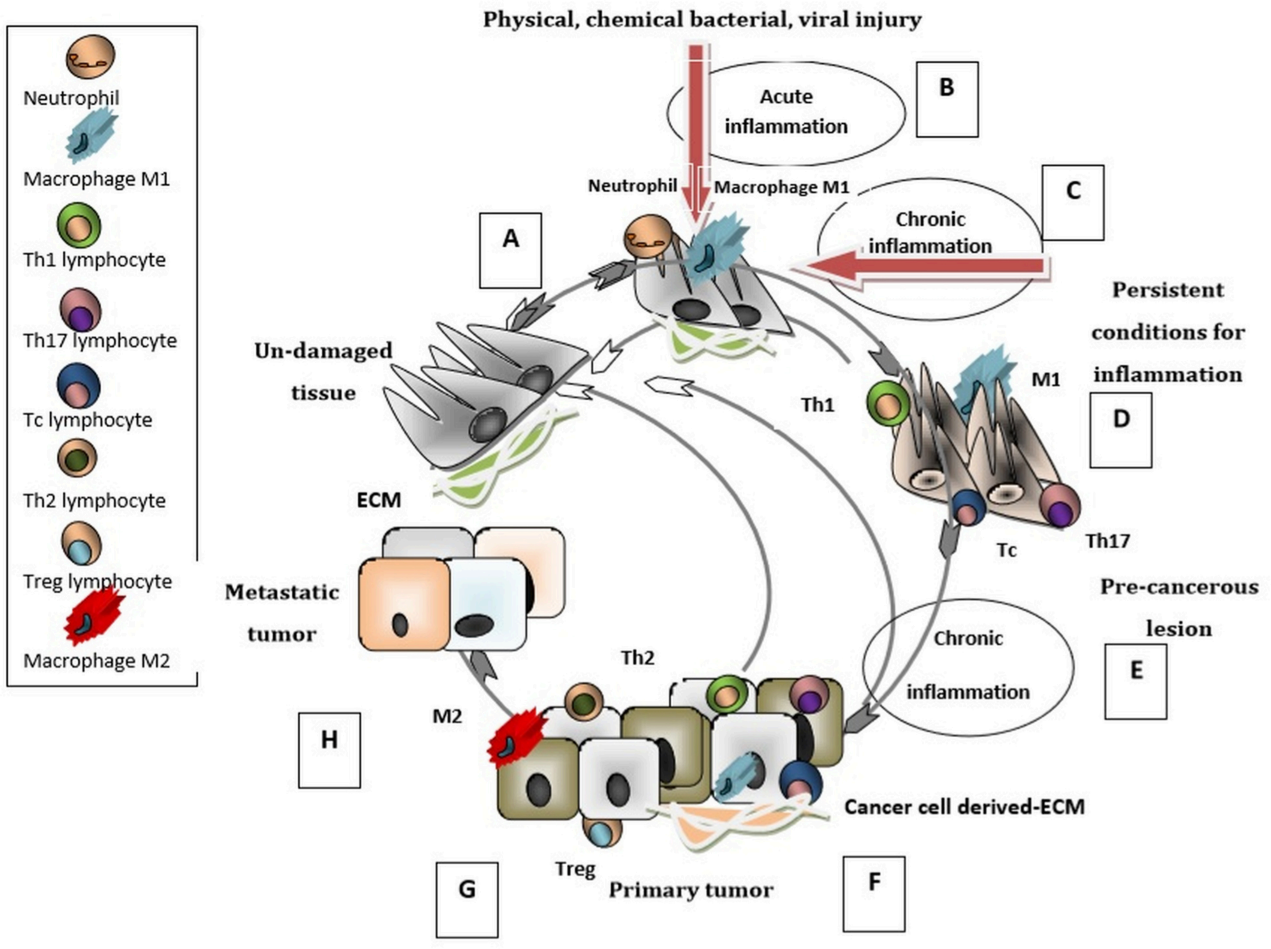
Immunobiology of cancer cycle. When un-damaged tissue **(A)** is subjected to injury, innate immunity cells (neutrophils and macrophages type M1) infiltrate the tissue and generate an acute inflammation **(B)** that triggers the repair cascade guiding the tissue to its un-damaged status. ECM is altered and contributes to inflammatory processes. If the inflammation is sustained and has chronic characteristics **(C)**, in addition to M1 macrophages the tissue is infiltrated with adaptive immunity cells (Th1, Tc, Th17); the pre-cancerous lesion **(D)** can return to its normal un-damaged status. At these stages the elimination of tumor development is possible. If the chronic inflammation persists **(E)** then the pre-cancerous lesion evolves into a primary tumor. The primary tumor can be infiltrated by M1, Th1, Tc, Th17 cells **(F)** with intense anti-tumoral activity which can restrain the tumorigenesis process and establish the immune equilibrium stage. In contrast, when the infiltrating cells are M2 type macrophages Th2, Treg lymphocytes **(G)** a pro-tumorigenesis milieu is enhanced and the primary tumor evolves into an aggressive/metastatic tumor. The ECM is modulated by tumor/stromal -cells and gains new immunosuppressive characteristics that favor metastasis **(H)**. This last stage of the tumor-immune cycle is characterized by the escape of tumor cells from the immune control.

The input of the immune system, introduced as cancer immunoediting, consists of three phases: elimination (i.e., cancer immunosurveillance), equilibrium, and escape (20). Importantly, the tumor ECM contributes to the development of an immunosuppressive network where stromal cells intertwine with inflammatory immune cells and with cells appending to the vascular system. Within this complex, a newly formed network, secreted cytokines and chemokines can sustain the tumor immune escape (21, 22). The chain of tumorigeneis can be initiated by injury of normal tissue, independently of the causative agent, and concomitant triggering of acute inflammation (23, 24). The maintenance of inflammatory conditions due to various effectors leads to chronic inflammation which may evolve to precancerous lesion (25–29). The evolvement of the pre-cancerous lesion can be attenuated by an active immune defense or driven to primary tumor development (30), as schematically presented in Figure 1. Thus, the immune system has the ability to perceive and destroy many tumors early on in their development, whereas during the stage of equilibrium, a restraint of the tumor is attained. Some tumors will succeed in escaping from the growth restriction maintained by the immune system, and become clinically apparent (20, 31).

The resolution of disease and response to therapy is likewise affected by the tumor microenvironment as two major subsets of tumors with distinct mechanisms of resistance to immune-mediated destruction have been recognized. Tumors exhibiting the inflamed immunophenotype present with the recruitment of CD8^+^ cytotoxic T cells, B cells and macrophages. Immune resistance in this case is due to the action of microenvironment-originating negative immune regulators. In non-inflamed tumors an absence of T-cells and innate immunity regulators is evident and leads to immediate immune failure (32, 33). Indeed, the ability of the tumor cells to escape impairment by the immune system has been suggested as a novel “hallmark of cancer” (6).

## Remodeling of the Cancer Environment

During tumor progression, an extensive remodeling of the ECM with correlated release of pro-tumorigenic factors and orchestration of surrounding “stroma” cells is initiated. This remodeling of the tumor microenvironment is closely associated to the modulation of the immune response (34, 35). Thus, in cancer pathogenesis the resulting “desmoplastic reaction” among resident fibroblasts, endothelial cells, pericytes, leukocytes and surrounding ECM is directly correlated with invasion and poor patient prognosis (36, 37). The restructuring of the ECM is due to: (i) modulation in the synthesis and release of ECM components; (ii) degradation of the ECM owing to enzyme action or chemical degradation due to radical oxygen species (ROS) (4, 38). Specific ECM remodeling will ultimately result in: (i) the detachment of tumor cells from each other, from adjacent stromal cells or from matrix; (ii) enhanced growth and mobility of tumor cells; (iii) modulation of the immune system and finally in sustaining of the tumorigenic microenvironment (39, 40).

## PGs are Active Mediators of Cancer-Associated Inflammatory Milieu

### Small Leucine Rich Proteoglycans Have a Dual Role in the Regulation of Cancer Inflammatory Setting

**Figure 2:**
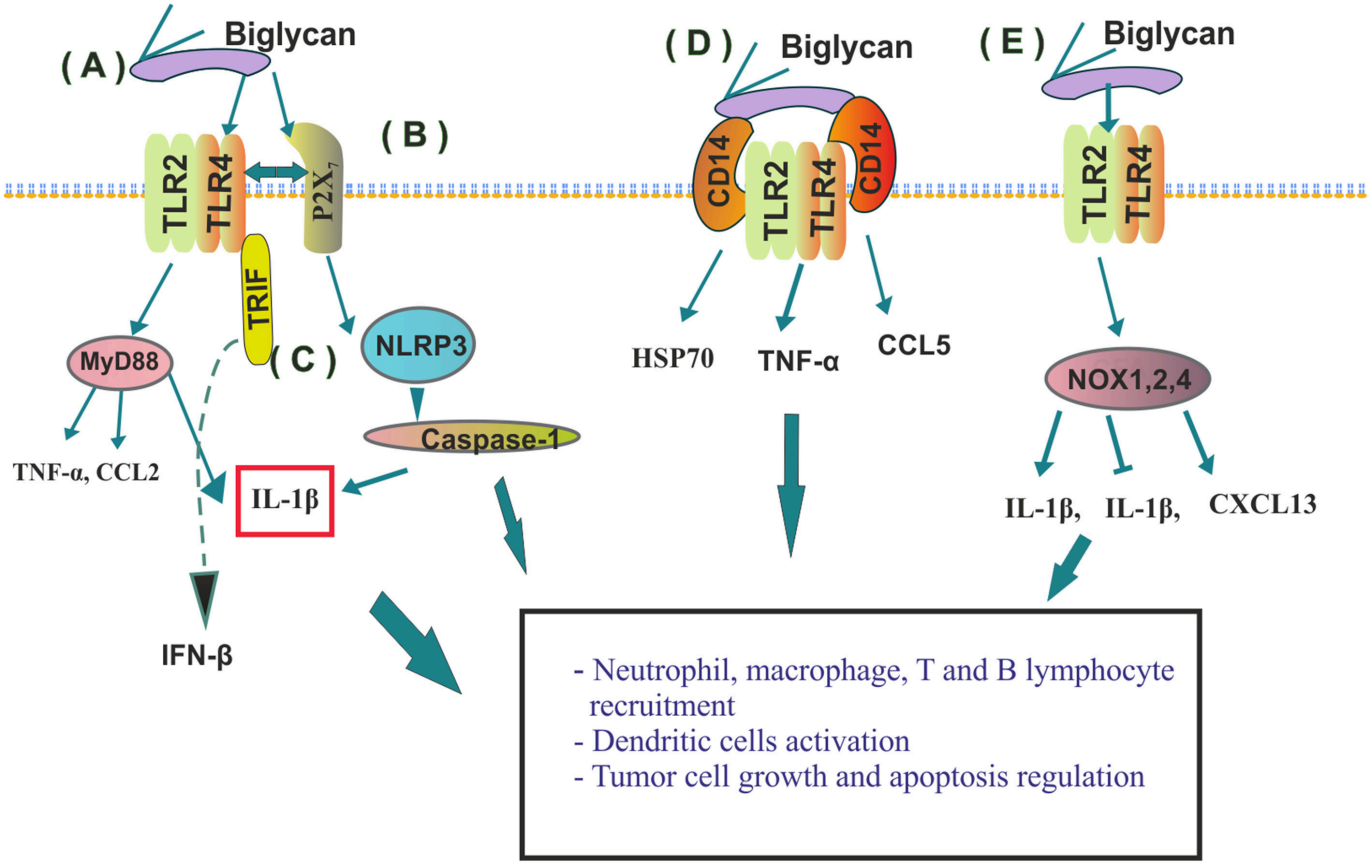
Immunomodulatory roles of biglycan. **(A)** By binding to TLR2 or 4 on macrophages' biglycan induces cytokine (TNF-α, CCL2 or IL-1β) release; **(B)** Crosstalk between TLR2/4- and P2X induces NLRP3 inflamozone and IL-1β activation and consequent neutrophil recruitment; **(C)** Soluble biglycan initiates Toll/interleukin (IL)-1R domain-containing adaptor activation inducing interferon (IFN)-β (TRIF) activities for T-lymphocyte recruitment; **(D)** Biglycans' binding determines CD14/TLR2/TLR4 complex formation and discrete downstream signaling; **(E)** Biglycan binding to specific TLR receptors discriminately activates NADPH oxidase (NOX) 1, 2, and 4 enzymes and regulates their downstream ROS production resulting in both positive or negative modulation of IL-1β synthesis; or in the stimulation of macrophages and dendritic cells to express chemokine (C-X-C) ligand 13 (CXCL13), the major chemoattractant for B and B1 lymphocytes.

The family of small leucine rich PGs (SLRPGs) was initially correlated with the regulation of innate immunological responses, noteworthy due to the fact that the triggering of these very responses can lead to the initiation of tumorigenesis (41). The role of a pro-inflammatory molecule has been designated to a class I SLRP member, biglycan (17). Importantly, this SLRP is overexpressed and secreted by various cancers including gastric (42), pancreatic (43), ovarian (44), and colon cancer (45). This SLRP can be either freed from the ECM through proteolytic degradation initiated upon tissue injury or *de novo* produced by activated macrophages and resident cells with various immunological roles (Figure 2) (46). Soluble biglycan can undertake the role of signaling mediator by binding to the Toll-like receptors (TLR)-2 and -4 on the surface of macrophages. The formation of the ligand-receptor complex initiates sterile inflammation and can facilitate pathogen-mediated inflammation through the production of pro-inflammatory cytokines and chemokines, including the tumor necrosis factor (TNF)-α, interleukin (IL)-1β or chemokine (C–C motif) ligand (CCL)2 (46, 47). It was shown that soluble biglycan utilizes TLR2/4 signaling pathways to activate adaptor molecule myeloid differentiation primary response 88 (MyD88), for the recruitment of neutrophils and macrophages or to initiate Toll/interleukin (IL)-1R domain-containing adaptor inducing interferon (IFN)-β (TRIF) activities for T-lymphocyte recruitment (48). Moreover, the role of a danger signal (DAMP) that triggers the NLRP3 inflammasome through upstream TLR2/4 and P2X receptors signaling has been attributed to biglycan (49), by specifically regulating the crosstalk between TLR2/4- and P2X_7_-NLRP3-caspase-1 (50, 51). Increasing data proposes that the initiation of TLR2/TLR4 signaling enhances tumor cell growth, downregulates apoptosis, and upregulates the synthesis of growth factors and inflammation-associated cytokines by tumor and stromal cells (50, 52, 53). Recently, biglycan was shown to bind with high affinity to macrophage CD14, an established GPI-anchored TLRs co-receptor. CD14 is mandatory for biglycan-dependent TLRs activation, where biglycan seems to have the role of a re-router as complexing with specific TLR members induces a discrete response. Thus, in macrophages, the biglycan/CD14/TLR2,4 complex induces the TNF-α expression, thebiglycan/CD14/TLR2 co-localization results in HSP70 release, whereas the biglycan/CD14/TLR4 complex initiates CCL5 secretion (54). Moreover, in a mouse model of renal injury, a deficiency of CD14 prevented biglycan-mediated cytokine expression, recruitment of macrophages, and M1 macrophage polarization (54).

Moreover, biglycan has been implicated in the regulation of important to tumorigenesis (4), ROS generation (55). Indeed, biglycan through activating discrete TLR receptors discriminately stabilizes NADPH oxidase (NOX) 1, 2, and 4 enzymes and regulates their downstream ROS production, resulting in either a positive or negative modulation of IL-1β synthesis (18). Furthermore, TLR2/4 induced ROS generation by biglycan (56) stimulates macrophages and dendritic cells to express chemokine (C-X-C) ligand 13 (CXCL13), the major chemoattractant for B and B1 lymphocytes (56). In addition to chemoattracting B cells, biglycan through TLR2/4 stimulates RANTES, MCP-1, and MIP-1α secretion, inducing macrophages and T cells recruitment into the kidney. Thus, these developments indicate that biglycan signaling can bridge innate and adaptive immunity (56), with the ability to act at different check points of the tumor immune cycle.

On the other hand, by enhancing NOX-generated ROS, biglycan induces genomic volatility coupled with chromosomal DNA modifications, which results in increased tumor cell growth, viability, and a metastatic ability of inflammation-associated tumors (57). Even though the majority of reports suggest that the activities of biglycan are pro-oncogenic, recent studies provide evidence that biglycan can promote an anti-inflammatory response that is key for the resolution of acute inflammation and hence the switch to acquired immunity response (18). Indeed, it is now proposed that biglycan, even though exhibiting affinity to both TLR2 and 4, will discriminately bind to only one TLR, which will, in combination with specific TLR adaptor molecules, lead to a discrete biological response (17, 18). The summarized regulation of immunity responses by biglycan can conceivably affect the development and resolution of early pre-cancerous lesions as well as the progression of inflamed tumor subtypes. Decorin, likewise an SLRP class I member, has established anti-tumorigenic properties (58). This SLRP member has the ability to initiate the TLR2/4 downstream signaling and cytokine release with an outcome different to that of biglycan. Specifically, utilizing a transforming growth factor –β (TGF-β)/oncogenic microRNA (miR)-21/tumor suppressor programmed cell death protein 4 (PDCD4) axis decorin downregulates the release of IL-10, which is an anti-inflammatory mediator and thus inhibits tumor growth (59). Moreover, decorin mobilizes mononuclear cells to the region of damaged tissue by enhancing CCL2 release and thereby, effectively upholding the inflammatory state (60). In a breast cancer xenographic model it was demonstrated that the decorin protein core inhibits genes obligatory for the regulation of the immunological response. Therefore, Buraschi et al. conclude that the “systemic administration of decorin protein core reveals a fundamental basis of action for decorin to modulate the tumor stroma as a biological mechanism for the ascribed anti-tumorigenic properties.” Indeed, the authors' gene ontology data indicated an inhibitory role in the regulation of proteins implicated in immunomodulatory responses when assigned to decorin protein core (61). The SLRP lumican has been postulated to modulate tumor-associated inflammation by affecting peripheral monocyte extravasation, and Fas–FasL signaling (14). Interestingly, lumican enhances LPS-dependent activation of TLR4 (62).

### The Hyalectan Versican Is Crucial to Both Innate and Adaptive Immunity

Versican, a member of the hyalectan family of large chondroitin sulfate PGs (CSPGs) has also been implicated in inflammatory processes (19, 63). These pericellular PGs are localized, among other, to the sub-endothelial compartment where they encounter the infiltrating leukocytes and modulate their biological activities by binding to specific receptors including TLR2, and P- or L-selectins (19). Once bound to the versican-containing ECM, leukocytes degrade the ECM to generate pro-inflammatory fragments that further drive the inflammatory response (64). Versican has been established to be overexpressed in a number of cancers, including prostate, breast, malignant myeloma, glioblastoma, laryngeal, pancreatic ovarian, gastric, testicular germ-cell, and cervical cancer, as recently discussed (65).

Importantly, in cancer-associated inflammation, versican can affect the production of cytokines by both lymphoid and myeloid cells. Thus, Lewis lung carcinoma (LLC) cells overexpress versican in that, by binding to TLR2 and its co-receptors on macrophage cell membranes, it activates the latter and facilitates their TNF-α secretion (66). The initiation of this cascade by versican and the induction of TNF-α by myeloid cells enhances LLC cell metastatic growth (66). Furthermore, an increased expression of versican V1 and V3 isoforms was positively correlated to lung metastasis in a bladder cancer murine model due to the increased release of CCL chemokine by macrophages (67). Specifically, versican was shown to facilitate bladder tumor cell migration, resulting in lung metastasis due to a mechanism involving CCL2/CCR2 secretion by macrophages. Said & Theodorescu propose that the aforementioned cytokine release generates a permissive lung inflammatory environment (68). Likewise, in mesotheliomas, versican downregulates macrophage M1 phenotype and decreases their ability to phagocytose tumor cells (69) contributing to the process of immune “escape.”

Interestingly, monocytes cultured in versican-containing supernatants of colon cancer cells secrete pro-inflammatory cytokines, including IL-12, TNFα and ROS, whereas monocytes cultured in versican-free supernatants gathered from breast cancer tumors' culture exhibited a different profile of secreted cytokines. Thus, versican seems to have the ability to specifically direct the inflammatory monocyte response (19), conceivably correlated to the differential recruitment of lymphocytes, and differentiation into tumor-associated macrophages and dendritic cells (DC) (70).

DCs are proposed to engage tumor antigens and relocate to draining lymph nodes, where they trigger tumor-specific T cells (71). Importantly, versican derived from tumor ECM activates DCs' TLR2, resulting in the production of immunosuppressive IL-10 and DC dysfunction. This was associated with a TLR2-induced increase of IL-6 and IL-10 cell-surface receptors and a strongly decreased cytokine concentration threshold is needed to trigger STAT3 (72, 73). Intact versican, thus, dampens DC activation (72, 73) and conceivably downstream Th and cytotoxic lymphocyte (CTL) differentiation. Likewise, intact versican secreted by macrophages was shown to have anti-inflammatory properties in a mouse model of acute pulmonary inflammation (74). On the other hand, in myelomas, versican is proteolyticaly processed to the DAMP versikine, which induces the secretion of IL-1β and IL-6 by human myeloma marrow-derived macrophages (MAMs). Importantly, MAMs chiefly synthesized V1, the precursor to versikine, whereas stromal cells secreted the versican-degrading protease. This interplay is suggested to enhance the “T-cell inflammation,” response and downregulate the “tolerogenic consequences of intact versican accumulation” contributing to tumor restraint (75).

### Syndecans, Modulators of the Inflammatory IL-6/STAT3 Pathway

Syndecans (SDCs), cell membrane HS containing PGs (76), have also been implicated in tumor immunomodulation. SDC1-defficient mouse model of colitis-related colon carcinoma exhibited higher susceptibleness to malignant transformation due to the enhanced topical release of IL-6, downstream activation of STAT3 and target genes with important roles in colonic tumorigenesis (77). Likewise, in inflammatory breast cancer, SDC1 was shown to upregulate the inflammatory IL-6/STAT3 pathway (78), a crucial part of tumor-stimulating signaling in epithelial-origin tumors (79). The well-described role of the enzyme heparanase, responsible for the cleavage of the HS chains may partly elucidate the role of the HS-bearing SDCs (80). Thus, due to the fact that HS regulates inflammatory processes at various checkpoints, e.g., including the segregation of inflammatory cytokines to the ECM, the configuration of leukocyte binding to the endothelial cells and ECM components, as well as the generation of responses respective to innate immunity by interacting with the TLRs, the reconfiguration of HS due to enzymatic heparanase affects the propagation of inflammatory events (80, 81).

### Endocan, A Unique Plasma PG Blocks Leukocyte Trafficking

Endocan is an endothelial-derived soluble PG, initially identified in human umbilical vein endothelial cells (82). Interestingly, endothelium activated by inflammatory processes and/or tumor pathogenesis exhibits a strong augmentation in endocan expression correlated to poor patient prognosis (81, 83–85). Glycanated human endocan has been shown to strongly promote tumor growth (86) through its ability to bind to the integrin CD11a/CD18 (LFA-1) and thus to block leukocyte binding to endothelium and subsequent infiltration to tumor tissues (87); or through its promotion of growth factor actions (83, 88). On the other hand, the non-glycanated mouse or human endocan polypeptide was shown, in a murine model, to delay tumor expansion through the induction of pan-leukocytic infiltration of CD122+ expressing cells within tumor and stroma tissues (89).

### The Intracellular PG, Serglycin, Is Implicated in Heamotological Malignancies

Serglycin, the only known intracellular PG is shown to be overexpressed in various tumor tissues and cell lines (90, 91). Importantly, serglycin is a key compound of secretory particles produced by CTL/NK cells, suggested to facilitate the safe storage of particle toxins, granzymes and perforin and affect CTL/NK cells' cytotoxic ability (92). Even though no evidence is available as such, these data implicate a plausible contribution of serglycin to processes of cancer-associated inflammation.

## Therapeutic Implications

The modulation of PGs' activities, as endogenous mediators of cancer-associated inflammation, is a novel approach in the field of cancer immunotherapy. Research efforts up to date have demonstrated the plausibility of this strategy as discussed below. Thus, the administration of the versican fragment characterized as DAMP, versikine, is suggested to “facilitate immune sensing of myeloma tumors and modulate the tolerogenic consequences of intact versican accumulation” which may facilitate tumor restraint and enhance T-cell-activating immunotherapies (75). Blocking the versican/CCL2/CCR2 signaling axis is indicated to purvey novel adjuvant strategies for detaining the emergence of clinical metastasis in bladder cancer patients (68). The non-glycanated mouse or human endocan polypeptide restrains tumor growth by increasing leukocyte infiltration *in vivo*, enhancing *de facto* innate immunity response (89). Suppressing the shedding of SDC1 from intestinal epithelial cells decreases intestinal inflammation and putative malignant transformation by augmenting NF-κB, respective downstream signaling, and neutrophil transmigration in ulcerative colitis (93). Indeed, the modulation of SDC1 expression could facilitate immunesurveillance and the positive resolution of precancerous lesions. Biglycan is suggested to bridge innate and adaptive immunity through TLR2/4 downstream signaling due to its' ability to regulate neutrophil, macrophage, T and B lymphocyte, and dendritic cells activities (17, 18, 46–56). Thus, selective inhibition of biglycan-TLR2/TLR4 axis could be a novel therapeutic approach targeting at various checkpoints of the cancer immunobiology cycle. Modulations of PGs post-transcriptional modifications, including their respective sulfation pattern, seems to be a therapy option as inhibition of sulfatase-2 activity had cytotoxic and partial hepatoprotective activity in both *in vivo* and *in vitro* hepatocellular carcinoma models (94, 95). Indeed, a clinically relevant pattern of PG and GAG expression and structural modifications was recently determined for PGs and GAG synthesizing enzymes in glioma and breast cancer, which might help in the development of personalized therapy (96).

Despite these advances, however, many issues need yet to be addressed for implementation to clinical practice (97). Furthermore, an understanding of tumor microenvironment biology and its PG component is necessary for therapy development.

## Author Contributions

DN designed the concept and completed the final editing of the manuscript. All authors contributed to writing of the manuscript. DN and MN prepared the figures. All authors read and approved the final version of the manuscript.

### Conflict of Interest Statement

The authors declare that the research was conducted in the absence of any commercial or financial relationships that could be construed as a potential conflict of interest.
